# An anatomy-based lumped parameter model of cerebrospinal venous circulation: can an extracranial anatomical change impact intracranial hemodynamics?

**DOI:** 10.1186/s12883-015-0352-y

**Published:** 2015-06-23

**Authors:** Stefania Marcotti, Lara Marchetti, Pietro Cecconi, Emiliano Votta, Gianfranco Beniamino Fiore, Antonello Barberio, Stefano Viotti, Alberto Redaelli, Maria Marcella Laganà

**Affiliations:** Department of Electronics, Information and Bioengineering, Politecnico di Milano, Piazza Leonardo Da Vinci, 32, 20133 Milan, Italy; Magnetic Resonance Laboratory, Fondazione Don Carlo Gnocchi ONLUS, IRCCS Santa Maria Nascente, Via Capecelatro 66, 20148 Milan, Italy

**Keywords:** Blood flow, Blood pressure, Cerebrovascular circulation, Nervous system, Veins

## Abstract

**Background:**

The relationship between extracranial venous system abnormalities and central nervous system disorders has been recently theorized. In this paper we delve into this hypothesis by modeling the venous drainage in brain and spinal column areas and simulating the intracranial flow changes due to extracranial morphological stenoses.

**Methods:**

A lumped parameter model of the cerebro-spinal venous drainage was created based on anatomical knowledge and vessels diameters and lengths taken from literature. Each vein was modeled as a hydraulic resistance, calculated through Poiseuille’s law. The inputs of the model were arterial flow rates of the intracranial, vertebral and lumbar districts. The effects of the obstruction of the main venous outflows were simulated. A database comprising 112 Multiple Sclerosis patients (Male/Female = 42/70; median age ± standard deviation = 43.7 ± 10.5 years) was retrospectively analyzed.

**Results:**

The flow rate of the main veins estimated with the model was similar to the measures of 21 healthy controls (Male/Female = 10/11; mean age ± standard deviation = 31 ± 11 years), obtained with a 1.5 T Magnetic Resonance scanner. The intracranial reflux topography predicted with the model in cases of internal jugular vein diameter reduction was similar to those observed in the patients with internal jugular vein obstacles.

**Conclusions:**

The proposed model can predict physiological and pathological behaviors with good fidelity. Despite the simplifications introduced in cerebrospinal venous circulation modeling, the key anatomical feature of the lumped parameter model allowed for a detailed analysis of the consequences of extracranial venous impairments on intracranial pressure and hemodynamics.

**Electronic supplementary material:**

The online version of this article (doi:10.1186/s12883-015-0352-y) contains supplementary material, which is available to authorized users.

## Background

The relationship between extracranial venous system abnormalities and central nervous system (CNS) disorders has been subjected to scrutiny in the latest years [1, 2]. In recent studies, researchers investigated the association of venous abnormalities with aging [3], leukoaraiosis [4] and neurological disorders like transient global amnesia [5–7], transient monocular blindness [8–10], exertional headache [11], multiple sclerosis [12] (MS) and Alzheimer's disease [13, 14]. Various mechanisms have been proposed to explain the role of venous abnormalities in CNS pathologies without a definite and final consensus of the scientific community. For instance, it was hypothesized that internal jugular veins (IJV) abnormalities (valve incompetence, reflux or stenosis) may cause intracranial hypertension. This fact would lead to modifications in cerebrospinal fluid dynamics, cerebral blood flow and cerebral blood perfusion; however the exact mechanism underlying these effects remains unknown [1].

Recently, a pathological vascular condition termed chronic cerebrospinal venous insufficiency (CCSVI) was introduced [12]. CCSVI was defined as the impairment of the main extracranial outflow routes, i.e., IJV, vertebral veins (VV), azygos vein (AZY). Hemodynamic and morphological criteria were proposed for its radiological diagnosis [12, 15]. The hypothesis assumed that CCSVI could have a causal relationship with MS [12], which has sparked significant debate in the literature [1, 16], as well as the criteria defining CCSVI itself and its diagnosis [15, 17].

Nevertheless, while even the concept of CCSVI itself remains highly contentious, the debate triggered interest in gaining a better understanding of the relationship between extracranial venous system abnormalities and CNS disorders [1]. Recent studies aimed at defining a protocol to examine the extra- and intracranial venous system *in vivo*, describing guidelines, limits, benefits, sensitivity and specificity of various non-invasive and invasive imaging methods [15, 17, 18].

In addition, biomechanical concepts were used to characterize the outflow from the brain through the main extracranial veins in physiological conditions and in subjects with occluded IJVs [19–21]. A hydrodynamic model of the main intracranial fluid flows [20], and a patient specific model [21] were proposed in order to explain how an extracranial alteration could produce intracranial impairment. However, a mathematical tool for modeling the principal extracranial and intracranial veins with anatomical fidelity and the analysis of their hemodynamic changes after morphological alterations are missing. Regarding the blood fluid-dynamics per se, different modeling techniques are available and have been previously adopted in physiological and pathological conditions with a particular focus on the arterial circulation due to the high mortality rate associated with arterial diseases. The venous models described in literature are often a part of more general models designed for whole body descriptions [22, 23], or for extracranial [19] or intracranial compartment only [24–26]. They were used for analyzing the global system instead of the local hemodynamics. The impact of IJV morphological alterations on the main intracranial vessels flow was recently described with a 3D model of the IJVs flow coupled with a 1D model of the main intracranial vessels [21]; however the smallest intracranial veins and the other extracranial routes are missing. For the same purpose, a model recently developed by Zamboni and colleagues [27] for studying the role of the collateral neck veins in the brain drainage was integrated [28] with the validated mathematical model for the simulation of the intracranial circulation of Ursino et al. [24].

In this work we propose a novel lumped parameter model of the venous cerebrospinal system based on anatomical knowledge of the venous network. This kind of modelization borrows concepts from electric circuit design: flows are modeled as currents, vessel resistance to the flow as impedance and pressure differences as voltage differences. We applied such model to quantitatively assess the relationship between local modifications in vessels lumen and the overall changes in drainage capability of the venous cerebrospinal system, with particular focus on the intracranial area. For this work, we considered subjects in the supine position, where the principal extracranial outflow routes are the IJVs, and the flow rate in the VVs is lower compared to the upright position [19, 28–32].

The flows estimated with the model were compared with those obtained with the phase contrast (PC) magnetic resonance images (MRI) from 21 healthy controls (HC).

The intracranial reflux location obtained simulating IJV obstruction was compared with EchoColor Doppler (ECD) examinations of 112 MS patients.

## Methods

### Cerebrospinal venous lumped parameter model

Hydraulic lumped-parameter models allow for quantifying pressures and flow rates at different locations within a vessel network in which different tracts can be described as combinations of i) hydraulic resistances relating the flow rate in a conduit and the corresponding pressure drop, ii) compliance elements quantifying the tendency of the conduit to dilate due to inner pressure, and iii) inertance elements accounting for the inertia of the fluid in the conduit.

In our case, compliance and inertance elements were neglected due to the fact that simulations were carried out in steady state. We modeled each vessel by means of a hydraulic resistance R, related to the vessel’s dimensions and blood rheological properties through Poiseuille's law:

1 $$ \mathrm{R}=\frac{128\upmu \mathrm{L}}{\uppi {\mathrm{D}}^4} $$

where L and D are the length and the equivalent diameter of the vessel and μ is blood viscosity, which was assumed equal to 3 cPoise [33].

Consistently, we assumed the standard linear relation between pressure drop (∆P) and flow rate (Q):

2 $$ \varDelta \mathrm{P}=\mathrm{R}\kern0.5em \cdot \kern0.5em \mathrm{Q} $$

Hydraulic resistances can be connected in series or parallel circuits: in the former case the total resistance is the sum of all single components, in the latter the reciprocal of the total resistance is the sum of the reciprocal of all single components. More complex networks can be built by combining these two elemental configurations.

**Fig. 1:**
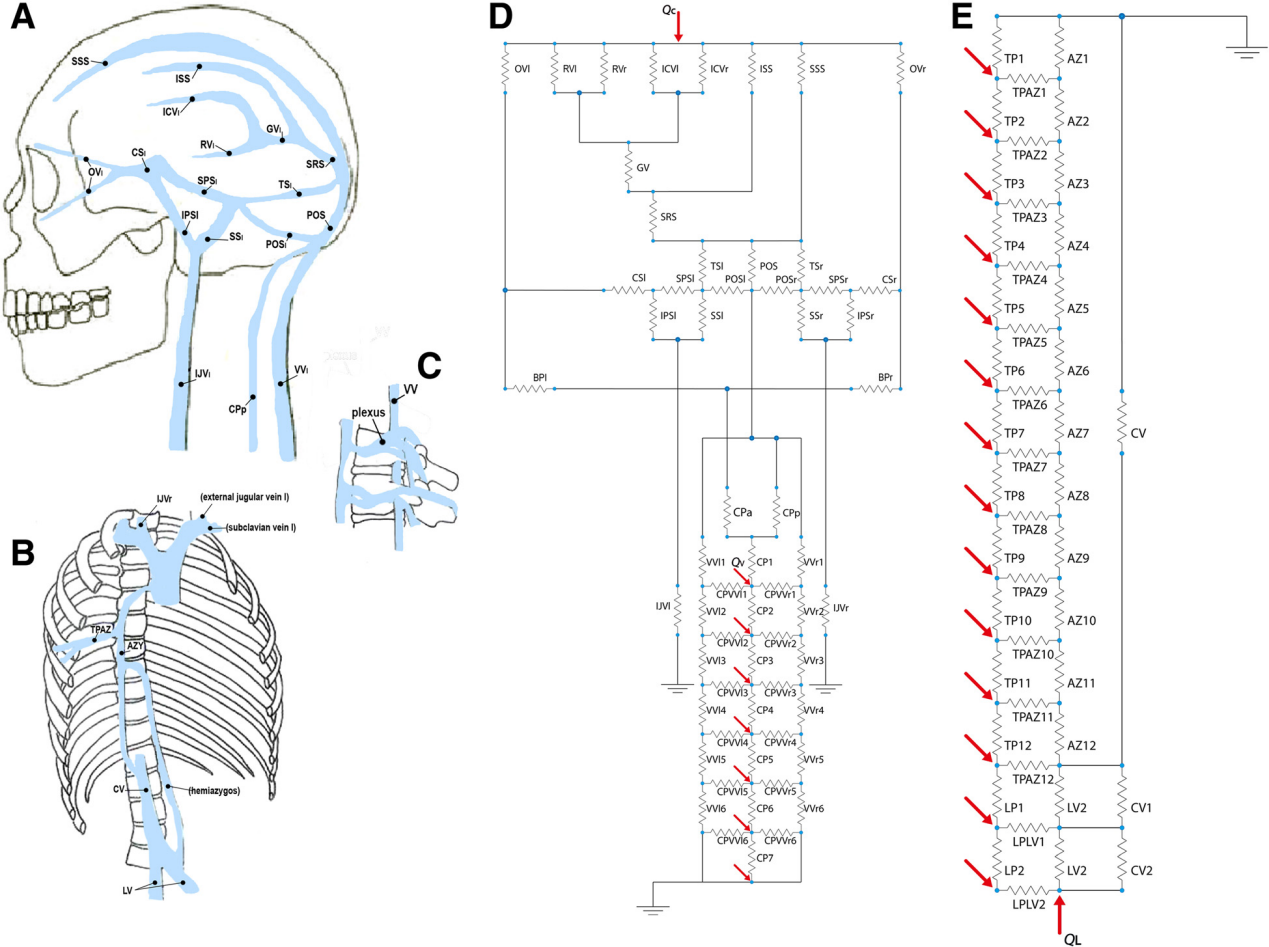
Cerebral (**a**, **d**), and extracranial (**b**, **c**, **e**) venous network: anatomical representation and lumped-parameter model. The 164 resistive elements (D and E) model 164 vessels. Flow inputs are indicated as arrows (Q_c_ at cerebral level, Q_f_ at femoral level, Q_v_ at vertebral level). The following venous vessels were included in the model, distinguishing between the right (subscript r) and left (subscript l) vessel whenever appropriate: for the intracranial area (**a**), ophthalmic veins (OV_l_, OV_r_), basal veins of Rosenthal (RV_l_, RV_r_), internal cerebral veins (ICV_l_, ICV_r_), inferior sagittal sinus (ISS), superior sagittal sinus (SSS), great vein of Galen (GV), straight sinus (SRS), transverse sinuses (TS_l_, TS_r_), posterior occipital sinuses (POS, POS_l_, POS_r_), basilar plexus (BA_l_, BA_r_), cavernous sinuses (CS_l_, CS_r_), superior petrosal sinuses (SPS_l_, SPS_r_), inferior petrosal sinuses (IPS_l_, IPS_r_), sigmoid sinuses (SS_l_, SS_r_); for the extracranial area (**a** and **b**), internal jugular veins (IJV_l_, IJV_r_), vertebral veins (divided into six segments, VV_l_ 1…6, VV_r_ 1…6), cervical plexus (divided into seven segments plus two, CP_a_, CP_p_, CP 1…7; subscripts a and p stand for anterior and posterior, respectively), connective vessels between cervical plexus and vertebral veins (CPVV_l_ 1…6, CPVV_r_ 1…6), thoracic plexus (divided into twelve segments, TP 1…12), azygos vein (divided into twelve segments, AZ 1…12), connective vessels between thoracic plexus and azygos vein (TPAZ 1…12), inferior vena cava (divided into three segments, CV, CV1, CV2), lumbar plexus (divided into two segments, LP1, LP2), lumbar vein (divided into two segments, LV1, LV2) and connective vessels between lumbar plexus and lumbar veins (LPLV1, LPLV2)

In order to model the main cerebrospinal veins, we considered 164 vessels and therefore 164 resistive elements, connected as shown in Fig. 1. The following venous vessels were included in the model, distinguishing between the right (subscript r) and left (subscript l) vessel whenever appropriate. For the intracranial area, ophthalmic veins (OV_l_, OV_r_), basal veins of Rosenthal (RV_l_, RV_r_), internal cerebral veins (ICV_l_, ICV_r_), inferior sagittal sinus (ISS), superior sagittal sinus (SSS), great vein of Galen (GV), straight sinus (SRS), transverse sinuses (TS_l_, TS_r_), posterior occipital sinuses (POS, POS_l_, POS_r_), basilar plexus (subdivided in right and left subportions, BP_l_, BP_r_), cavernous sinuses (CS_l_, CS_r_), superior petrosal sinuses (SPS_l_, SPS_r_), inferior petrosal sinuses (IPS_l_, IPS_r_), sigmoid sinuses (SS_l_, SS_r_). For the extracranial area, internal jugular veins (IJV_l_, IJV_r_), vertebral veins (divided into six segments, VV_l_ 1…6, VV_r_ 1…6), cervical plexus (divided into seven segments CP 1…7 and anterior and posterior segments, CP_a_, CP_p_), connective vessels between cervical plexus and vertebral veins (CPVV_l_ 1…6, CPVV_r_ 1…6), thoracic plexus (divided into twelve segments, TP 1…12), azygos vein (divided into twelve segments, AZ 1…12), connective vessels between thoracic plexus and azygos vein (TPAZ 1…12), inferior vena cava (divided into three segments, CV, CV1, CV2), lumbar plexus (divided into two segments, LP1, LP2), lumbar vein (divided into two segments, LV1, LV2) and connective vessels between lumbar plexus and lumbar veins (LPLV1, LPLV2). Facial venous circulation was excluded from the model, as well as external jugular veins, which drain the extra-cranium area. With regards the intra-cranium compartment, minor vessels such as venules were neglected.

Vessels dimensions (Additional file 1) and configuration were set based on the physiological anatomy of venous drainage [31, 34–37] for a subject in the supine position.

Inputs of the system were arterial flows of cerebral (Q_c_), femoral (Q_f_) and vertebral (Q_v1_…Q_v21_) regions. With regards to the vertebral area, a single Q_vi_ was considered for the i-th vertebra. Pressure was set equal to zero at two points of the hydraulic system: i) at the level of subclavian vein, based on its notably higher cross sectional diameter, and hence minimal hydraulic resistance, compared to the other vessels included in the model (Equation 1), and ii) at the level of the right atrium, consistent with negligible atrial pressure in physiological conditions. Based on the latter assumption, the hydraulic system was divided into two decoupled parts; the first representing the cerebral and upper spinal areas, and the second representing the cardiac area and the remaining part of the spinal column. This subdivision implied independent inputs at cervical (Q_c_ and Q_vi_, i=1,…, 7) and thoracic/lumbar (Q_f_ and Q_vi_, i=8,…, 21) levels.

We computed pressures (Pa) at each node and flow rates (ml/s) for each vessel by solving the linear system of equations associated with the network through a Matlab® script (The MathWorks Inc., Natick, MA, United States).

### MRI acquisition and processing

Twenty-one HC (Male/Female = 10/11; mean age ± standard deviation = 31 ± 11 years) were recruited for measuring normal flow rates in the main cerebrospinal venous vessels with PC MRI. Data were acquired using a 1.5 T Siemens Magnetom Avanto scanner equipped with a 12-channel head coil and a 4-channel neck coil at IRCCS S. Maria Nascente of Fondazione Don Carlo Gnocchi ONLUS, Milan, Italy. The study was approved by the Don Gnocchi Foundation Ethics Committee (Milan, Italy) and written informed consent was obtained from all subjects prior to study entry.

The following sequences were acquired: 1) T1 weighted localizer of the brain and of the neck (three perpendicular slices) for the slice positioning of subsequent sequences; 2) dual-echo turbo spin echo (TR=2650 ms, TE=28/113 ms; echo train length=5; flip angle=150°, 50 interleaved, 2.5-mm-thick axial slices, with a matrix size=256×256, interpolated to 512×512, and a FOV=250×250 mm^2^) for the exclusion of vascular pathology, or white matter hyperintensities; 3) 2D time-of-flight magnetic resonance venography (TOF MRV) of the head and neck (TR=21 ms, TE=4.78 ms, 128 axial slices, FoV= 320×255 mm^2^, voxel of 1×0.5×3 mm^3^, distance factor of −20 %), with a caudal saturation band, in order to saturate the arterial signal, for positioning the following sequences; 4) 2D PC sequences for the flow quantification of cerebrospinal venous vessels, as follows. For the IJVs and the VVs, a PC slice was positioned between the fifth and the sixth cervical vertebrae (C5-C6), and between C2-C3, respectively. The IJVs were scanned with a slice positioned at the cervical level between C5 and C6, the VV were scanned with a slice at the cervical level between C2 and C3. Other vessels acquired, when visible, were: ISS, SRS, left and right RV, ICV, TS, SPS, SS, anterior and posterior portions of SSS. Their anatomical positions were identified on the proton density weighted images, or on TOF sagittal or coronal maximum intensity projection. The PC slices were positioned perpendicular to the axis of considered vessel. The maximum encoding velocity was selected depending on the expected maximum velocity of the examined vessel: 60 cm/s for IJVs and VV; 50 cm/s for SRS, TS and SSS; 20 cm/s for the ISS, ICV and R, and SPS. All the PC sequences were acquired with a retrospective cardiac gating through a finger pulse oxymeter, allowing the reconstruction of 25 time points over the cardiac cycle.

The PC data were processed with Argus software (Siemens Medical Solutions): for every vein and every time point, the vessel contour was manually drawn on the magnitude image and then transferred onto the corresponding phase image. The phase offset, i.e., the phase value of stationary structures, was estimated as the mean phase value inside a region of interest drawn in the muscular tissue. For every pixel inside the segmented vessels, and for every time point, the phase value, corrected by the offset, was mapped to velocity. From the area of the segmented vessel and the velocity in each pixel, the average flow rate (in ml/s) over the cardiac cycle was computed.

### Comparison between flow rates computed with physiological model and MRI

**Fig. 2:**
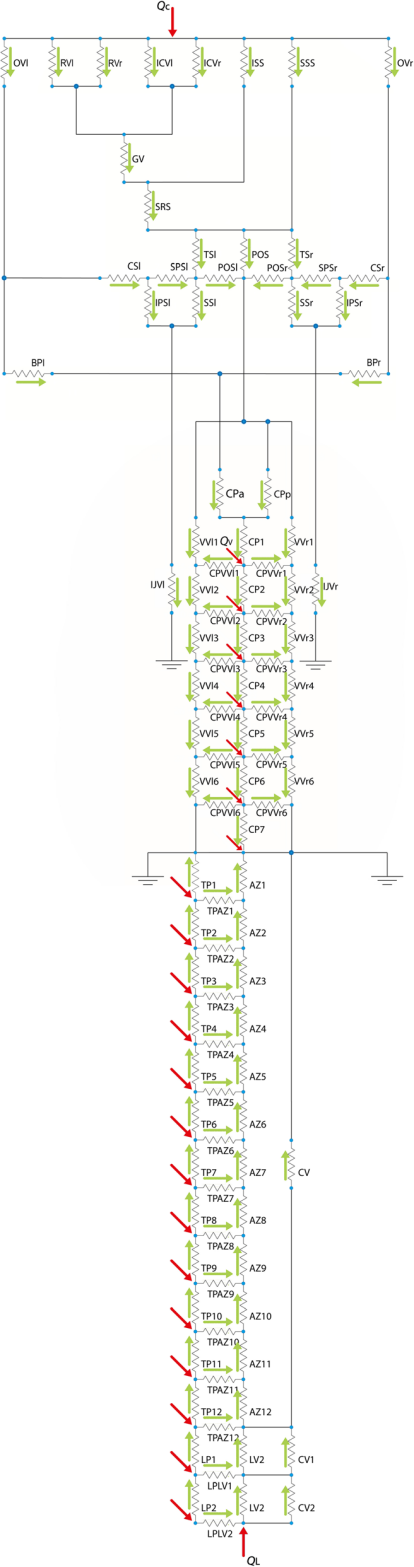
Flow directions computed by the model for the cerebral and extracranial areas. Flow directions of each vessel of the network are shown as arrows

The flow direction in each modeled vessel was compared with known physiology (Fig. 2).

The one-sample Wilcoxon signed rank test was used to assess whether the mean flow rate measured with PC MRI in HC differed significantly from the model estimate. For the VVs, we compared the flow rates measured with MRI with rates computed with the model at the second VV segment. A p-value <0.05 was deemed significant while <0.1 was considered a trend.

### Venous obstruction simulation

The effect of the main outflow routes obstruction was simulated with the model. The following cases were implemented: monolateral and bilateral obstruction of the internal jugular veins, the vertebral veins and the proximal azygos. In details, our analysis was performed as following.

#### Obstruction of the IJV

In order to simulate partial or total IJV occlusion, the diameter of each IJV was reduced from 0 % (complete patency) to 100 % (complete block), with increments of 10 %. For each of the 121 simulations, we computed the flow rates of each intracranial vein and the pressure at each intracranial node and at IJV upstream. We also checked which kind of bilateral IJV obstruction produced intracranial retrograde flows.

We repeated the same analysis with other published physiological IJV diameters: 1 cm [29] and 0.7 cm [32].

Moreover, the analysis was carried out adding collateral vessels [38, 39] in parallel with the IJVs, using their same length, each with a diameter of 0.4 cm each (Additional file 1).

#### Vertebral vein obstruction

The diameter of all the VV segments was reduced from 0 to 100 %, with increments of 10 %, to simulate their partial or total obstruction. For each configuration, we assessed pressures and flow rates of each vessel and when and where the reflux was produced.

#### Azygos obstruction

The diameters of the first four proximal azygos segments were reduced from 0 to 100 % with increments of 10 %. For each configuration, we assessed the pressure of each azygos portion and checked if any azygos or thoracic plexus segment was refluent.

### Echodoppler acquisition

The ECD of 112 MS patients were acquired with the ultrasound system MyLabVinco (Esaote S.p.A., Firenze, Italy) by an expert vascular sonographer. Fourty-nine patients were examined as part of a previously published multicenter study [40] whereas the others were acquired during clinical examinations. An overview of the demographic and the clinical characteristics of all the MS patients are detailed in Table 1.

**Table 1:** Demographic and clinical characteristics of multiple sclerosis patients.

	All MS	RR MS	SP MS	PP MS
N	112	63	45	4
age (years)	43.7±10.5	40.4±9.3	47.1±10	56.8±13.8
age at disease onset (years)	32.5±10	31.5±9.4	33±9.5	44.7±21.2
disease duration (years)	11.4±8.2	9.4±8.7	14.4±6.7	9.7±5.5
EDSS	5 [0.0 – 7.5]	2 [0.0 – 6.5]	6.5 [2.0 – 7.5]	6 [5.0 – 6.5]
M/F	42/70	23/40	17/28	2/2

Age at time of Doppler evaluation, age at disease onset, disease duration at time of Doppler evaluation, Expanded Disability Status Scale (EDSS), and number of male and female subjects. Mean ± standard deviation of age and disease duration are expressed in years; EDSS is expressed as median [range]

The ultrasound system was equipped with a linear array transducer probe (LA332, Esaote SpA, Italy: operating bandwidth 3 – 11 MHz; imaging frequencies: 3.5 – 5.0 – 6.6 – 10.0 MHz; Doppler frequencies: 3.3 – 5.0 MHz) for the IJV and VV examination and with a phased array transducer probe (PA240, Esaote SpA, Italy: operating bandwidth: 1–4 MHz; B-Mode frequencies: 2.0 – 2.5 – 3.3 MHz) for intracranial examination. A thick layer of US gel (Aquasonic 100 – Parker Laboratories Inc, Fairfield, New Jersey USA) was used to ensure a complete coupling between the transducer and the examined subject’s skin, to avoid black cones and dark areas on the US image.

During the IJV examination, the sonographer paid particular attention to prevent excessive pressure on the examined subject’s neck, in order not to change IJV shape and dimension. B-mode was used to assess flow obstruction due to stenosis, annulus, or septums.

The intracranial examination was performed through the temporal and transcondylar windows in order to assess the flow direction of RV and petrosal sinuses, respectively [41]. The Multigate Quality Doppler Profiles was also used to detect flows with slow velocity or different directions in the same vessel and to detect intracranial reflux [42].

While the full protocol requires examining the subject in supine and sitting positions, we considered only the data obtained in the supine examination for this work.

The occurrence and topography of venous reflux observed through ECD examinations with the contemporaneous presence of mono- or bilateral IJV obstruction was compared with estimates from the model.

## Results

### Comparison between model estimates and MRI measures

The model correctly reproduced local flow direction in all its segments (Fig. 2).

**Fig. 3:**
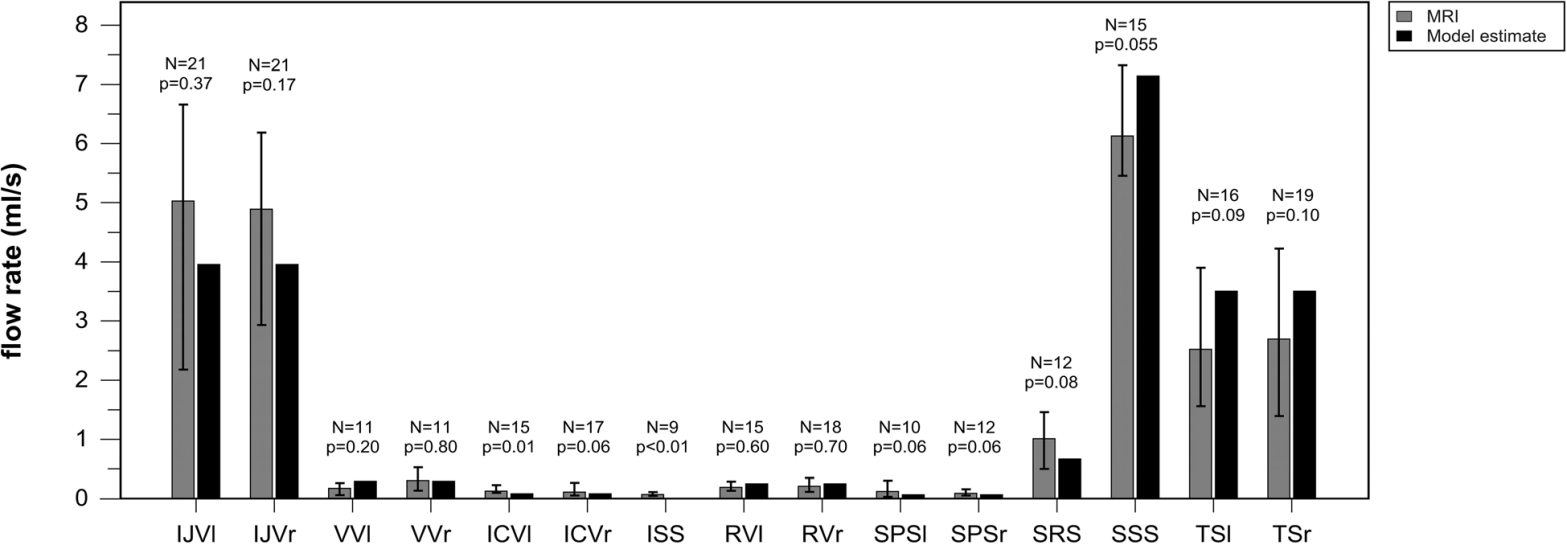
Comparison between physiological model estimates and flow rate quantified with PC MRI in healthy controls (HC). The median and 95 % confidence interval are shown for the MRI measurements. The number of correctly imaged vessels and the p-value of the one-sample Wilcoxon signed rank test are reported over the corresponding vessel. The following vessels are shown: for the extracranial area, internal jugular veins (IJV_l_, IJV_r_) and vertebral veins (VV_r_, VV_l_); for the intracranial area, internal cerebral veins (ICV_l_, ICV_r_), inferior sagittal sinus (ISS), Rosenthal veins (RV_r_, RV_l_), superior petrosal sinuses (SPS_r_, SPS_l_), straight sinus (SRS), superior sagittal sinus (SSS, values obtained with the posterior acquisition), transverse sinuses (TS_r_, TS_l_)

Figure 3 shows the comparison between the flow rate measured with PC MRI and the corresponding model estimate. The number of vessels correctly imaged and the p-value of the signed rank test are also reported. We did not measure SS_r_ or SS_l_ flow rates as it was not possible to correctly image these vessels solely in the perpendicular plane. The flow rate measured with MRI was not significantly different from the model estimate for the following veins: IJV_r_, IJV_l_, VV_r_, VV_l_, ICV_r_, RV_r_, RV_l_, SPS_l_, SPS_r_, SRS, posterior part of SSS, TS_l_, TS_r_. The flow rates of ISS, ICV_l_ and anterior SSS estimated with the model were statistically different from those measured with MRI. The median flow rate obtained with MRI for anterior SSS (not shown in Fig. 3) and its 95 % CI were 2.5 [1.1–3.1] ml/s, which is statistically different (p<0.0001) from that estimated by the model (7.1 ml/s).

### Venous obstruction simulation

The structure of the lumped parameter set allowed for separate analysis of the effects of upper and lower circulation anatomical anomalies. As the upper and lower parts of the model were decoupled, changes in the portion above the cardiac level did not affect the one below, and vice versa.

#### IJV obstruction

**Fig. 4:**
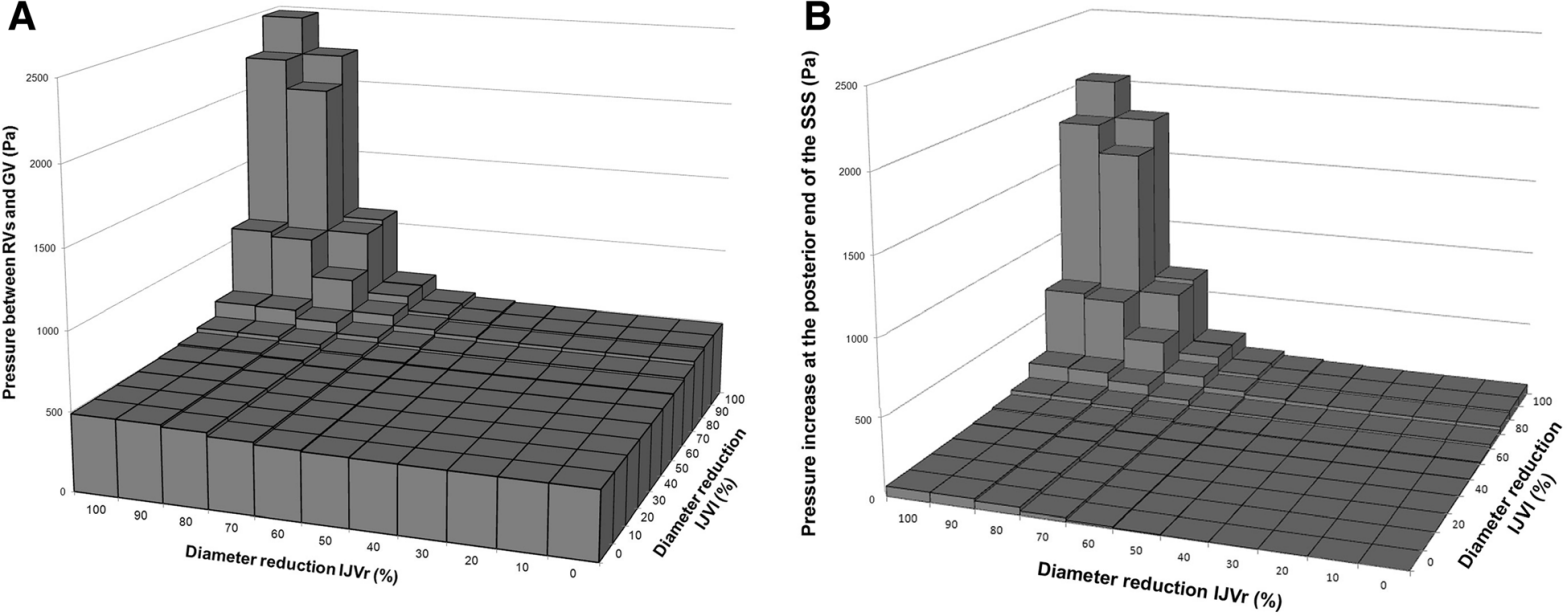
Intracranial pressure obtained with model simulations of internal jugular veins (IJVs) (**a**, **b**) diameter reduction. **a** Pressure at the confluence of Rosenthal veins (RVs) to Galen vein (GV) *vs* internal jugular veins (IJVs) diameter reduction. The result is symmetric for correspondent diameter reduction rates on the opposite side (not shown). Pressure doubled its initial value when one of the IJV had a diameter reduction of at least 80 % and the other one of at least 90 %. **b** Difference between the SSS pressure estimated with patent IJVs and the SSS pressure estimated reducing IJVs diameter

The intracranial pressure estimated by the model had a specific trend related with the degree of IJV diameter reduction as shown in Fig. 4a. The amplitude depends on the specific node. Figure 4a provides, as an example, the pressure at the confluence of RVs to GV. At this level, intracranial pressure doubled its baseline value when both the IJVs were obstructed, in particular when one of them had a diameter reduction of at least 80 % and the other one of at least 90 %. Fig. 4b shows how the SSS posterior end pressure increases with different IJVs constrictions, with respect to the baseline value, which was removed from each value.

The simulations carried out with IJV diameters of 1 cm [29] and 0.7 cm [32] showed intracranial pressure increase for lower IJV diameter reductions. At the level of the RVs confluence to GV, pressure doubled its baseline value for bilateral IJVs obstruction of 70 % in the latter and 60 % in the former (Additional files 2 and 3) case.

In regards to the IJV upstream pressure, it doubled its initial value for IJV diameter reduction of at least 20 %.

When the total occlusion of one IJV was simulated, retrograde flow in the corresponding SS was computed whenever a partial diameter reduction of the other IJV was modeled.

**Fig. 5:**
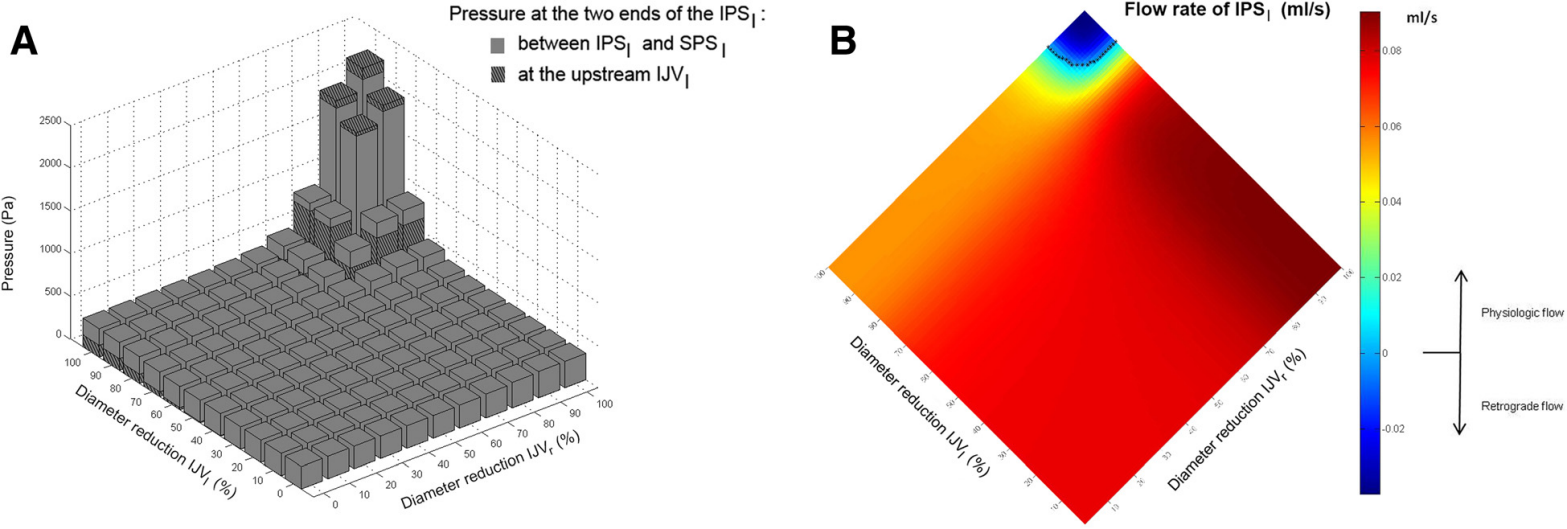
Pressures at the IPS_l_ ends (**a**) and IPS_l_ flow rates (**b**) obtained with model simulations of internal jugular veins (IJVs) (**a**, **b**) diameter reduction. **a** Pressure between the IPS_l_ and the SPS_l_ (distal extremity of IPS; uniform gray bars) and pressure at the upstream IJV_l_ (proximal extremity of IPS; non-uniform gray bars) are superimposed on each other. **b** IPS_l_ flow rate, estimated at different IJVs diameter reduction, with increments of 1 %. Matlab jet colormap was used in order to clearly discern positive and negative values, that are physiologic and retrograde flows. The zero-level is highlighted with asterisks

The simulation of bilateral IJV occlusion greater than 90 % produced retrograde flow in the CS, IPS (Fig. 5b) and SPS. The simulations carried out with IJV diameters of 1 cm [29] and 0.7 cm [32] showed reflux of intracranial vessels for lower percentage of bilateral IJV diameter reduction (80 % in the former and of 70 % in the latter case).

The results obtained with IJV collaterals differed from those obtained without collaterals by less than 3.5 %. The reflux was observed in the same vessels as without collaterals.

#### Vertebral vein obstruction

The simulation of VV diameter reduction showed an increase in global high pressure in the cervical and vertebral areas, with higher values for severe VVs obstruction. Conversely, the intracranial area was less affected, where the pressure increases by less than 14 %. The CP_a_ and CP_p_ upstream, respectively the confluences of the BP and the OS to the cervical and vertebral areas, were highly affected by VV obstruction.

Retrograde flow in POS, POS_l_ and POS_r_ was observed for bilateral VV occlusion higher than 60 %.

#### Azygos obstruction

**Fig. 6:**
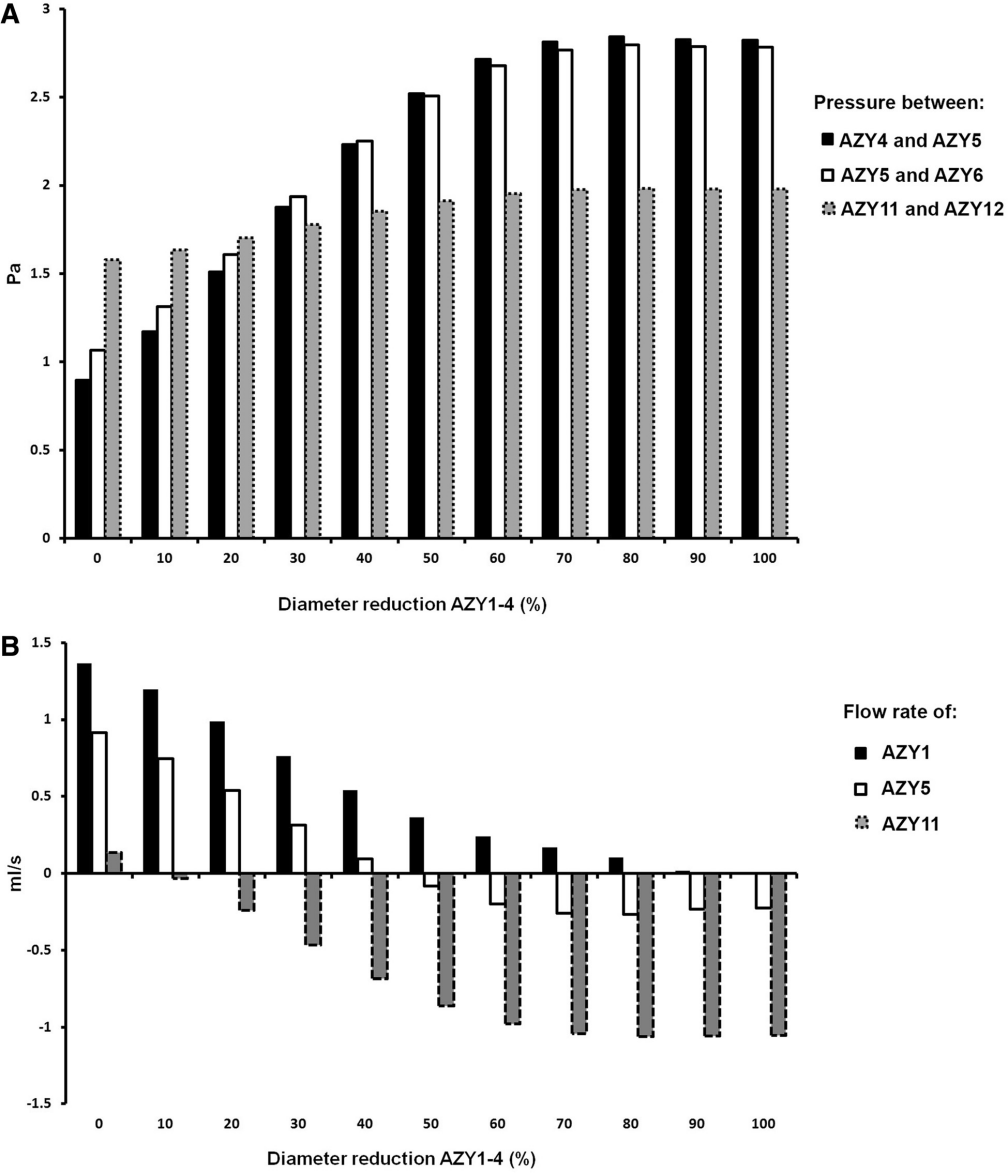
Results obtained with model simulations of azygos (**a**, **b**) diameter reduction. **a** Bars showing the increase of the pressure at the node between the fourth and the fifth azygos, the fifth and the sixth, and the eleventh and twelfth azygos portions vs diameter reduction of the first four proximal azygos segments. **b** Flow rate of AZY1, AZY5, and AZY11 versus diameter reduction of the first four proximal azygos segments. The flow and the pressures at the ends of AZY5 are represented as exemplificative: its flow is retrograde when we simulated a diameter reduction higher than 50 % for the first four AZY segments, because the proximal pressure begins to exceed that of the distal end

The obstruction of the proximal azygos segments caused a pressure increment along the azygos. The pressure progressively increased as the azygos diameter was reduced. The obstruction effect was higher for the nodes near to the obstruction itself and lower for the distal parts. We reported (Fig. 6a) the pressure between AZY4 and AZY5, and AZY5 and AZY6 as representative of the former, and the one between AZY11 and AZY12 for the latter.

Retrograde flows were computed in the azygos itself and in the thoracic plexus: the higher the degree of diameter reduction, the higher the number of distal azygos and thoracic plexus segments affected by the reflux. Figure 6b shows the flow rate trend with progressive azygos diameter reduction for AZY1, AZY5, and AZY11, as representative of segments from proximal to distal azygos.

### EchoColor Doppler results

ECD data of MS patients showed IJV abnormalities caused by stenosis, membranous obstruction or hypoplasia in 86 % of cases. Among the stenotic or hypoplastic cases, unilateral IJV occlusion was detected in 28 patients (21 of them had unilateral IJV but not VV occlusion), while bilateral in 37 (17 without VV occlusions). Intracranial reflux was observed in 21/28 subjects with monolateral IJV occlusion (17/21 considering those without VV occlusions), in 28/37 subjects with bilateral IJV occlusion (15/17 considering those without VV occlusions). The reflux was always located in CS, IPS and SPS, but never located in the RV.

## Discussion

In the present work we described a lumped parameter model of the cerebrospinal venous network based on a realistic anatomical paradigm, aimed at determining if an extracranial morphological change can cause an intracranial venous flow alteration.

Compared to previously published models of the physiologic and impaired cerebrospinal venous circulation [19, 21, 23, 25, 26], our model was designed with a detailed correspondence between its elements and anatomy. The accuracy of the model was evaluated comparing the flow estimates with that obtained with PC-MRI from a group of healthy subjects.

We applied the model to mimic obstruction of the main extracranial veins and to estimate the changes in intracranial pressure and flow. We compared the location of flow inversion simulated with IJV obstruction and what we observed with ECD in a group of 112 MS patients.

### Comparison between model estimates and MRI measures

The physiologic flow rates computed by the model were realistic in terms of direction (Fig. 2) and amplitudes (Fig. 3). These agreements were not obvious since we a priori set the arterial input flow directions; the network connections; the venous diameters and lengths and the zero-level pressure at two points of the hydraulic system. We did not impose the venous flow directions or pressures, with the exception of the pressures in subclavian vein and the right atrium. In regards to the flow amplitude, the mean flow rates estimated with MRI were not significantly different from those predicted by the model, with the exception of ISS and ICV_l_.

The best correspondence between predicted and measured flow rates were found for those vessels whose anatomy can be approximated by an almost straight pipe of constant cross sectional area, i.e., where the basic assumption for Poiseuille's law holds, and whose dimension, shape and blood velocity allowed for the acquisition of good quality PC MRI data.

The significant difference between ISS (p=0.004) and ICV_l_ (p=0.01) flow rates and those predicted by the model could be due to the small dimensions and low flow velocity of all these vessels and to the curve shape of the ISS. The same reasons could explain the trend for difference found between ICV_r_ and both the SPS (p=0.06).

Regarding the SSS, its cross sectional dimensions increased anteriorly to posteriorly; as did its flow rate, consistently with the presence of the superior cerebral veins converging into it. We modeled the SSS as a single resistance and did not model the superior cerebral veins due to their high inter- subject variability in terms of number and size. For these matters the flow rate measured with the anterior, but not posterior, acquisition was significantly different from the model estimate. Furthermore, the SSS curve shape could explain the trend for difference between the model estimate and the posterior MRI measurement (p=0.055). The mean SSS flow rate measured with the posterior acquisition was similar to that reported by Gideon et al. [43]. (443±124 ml/min, p=0.1) and Mehta et al. [44] (388±169 ml/min, p=0.9) on a group of 14 and 15 healthy volunteers, respectively. The model estimate was similar to the cited works. The same reasons could explain why there was a trend for the TS (p=0.09 and p=0.1for the left and the right side, respectively) for slower flow rates measured with MRI compared to that predicted by the model.

Another general source of mismatch between our model estimates and the MRI measurements involves the coupled vessels. We assumed a right-left symmetry of the venous network when designing the model, while *in vivo* asymmetry is described with a wide range of anatomic patterns [45–49]. The IJV, VV, and TS, seem to have right dominance regarding their size and flow [47, 49].

As a matter of principle, different anatomical connections could be additionally implemented with the same approach, even though this would require changing the equations associated to the network. The same model structure can instead be used for a variety of analyses, such as the study of asymmetric vessels, only by changing the dimensions of the vessels.

### Venous obstruction simulation

The simulations obtained using different combinations of IJV diameters showed that the intracranial pressure is stable for a wide range of IJVs diameters and increases for severe IJV stenosis. Our finding is similar to that showed by the recent model of Gadda [28], where a mild effect on the venous sinuses pressure was produced by halving the IJVs conductances, and a strong effect by setting the IJVs conductances close to zero. The previously cited model analyzed the venous sinuses pressure as a single node of the network. Conversely, our model includes the main intracranial vessels, so we could simulate the pressures at their ends for progressive bilateral IJV constrictions. Even with different amplitudes, the relationship between venous pressure and IJV diameter reduction had the same shape for the different intracranial veins. The high pressures estimated at the confluence of RVs and ICVs to GV due to severe IJVs diameter reduction is a theoretical biomechanical finding supporting the hypothesis and the experimental findings of previous works [20, 50–52]. It has been suggested that such a high pressure can damage the brain tissue, with possible blood–brain-barrier rupture [50, 51] or permeability increase [13, 53]. In particular, since the Galenic system drains the periventricular white matter and the basal ganglia, its increase of pressure can damage those brain areas [51, 53]. The intracranial vein hypertension, especially that of the SSS (Fig. 4b), can also impact the cerebrospinal fluid flow hemodynamics. Indeed, the cerebrospinal fluid flow absorption is possible if cerebrospinal fluid pressure exceeds that of the venous sinuses by about 5–7 mmHg [20]. For severe degrees of IJV stenosis, our model predicted a pressure increase in the SSS similar or even higher than the previously reported pressure gradient. A relationship between IJV stenosis, reflux and cerebrospinal fluid flow alterations was recently observed in different CNS disorders, like MS and AD [13, 14, 20, 54, 55]. The cerebrospinal fluid flow alterations were also recently reported to correlate with “dirty appearing white matter” in healthy subjects [56]. The brain edema seen in cases of venous sinus thrombosis or stenosis could also be due to the decrease in cerebrospinal fluid drainage caused by an increase in venous pressure [57]. In a recent review [20], Beggs explained the link between IJV stenosis, its hypertension and the impairment of cerebrospinal fluid flow absorption into the venous blood.

Other consequences of severe neck vein stenoses predicted by our model are the reduced intracranial venous flow rate, and even flow inversion. Schelling in 1986 [52] suggested that refluent IJVs or intracranial veins could produce retrograde venous hypertension, i.e., from downstream to upstream the venous network. The resulting venous overload, engorgement, dilatation and leakage were proposed to be possible causes of MS lesions. The location and shape of such lesions were also suggested to be similar to those found through Schlesinger’s experiments with gelatin solution forced in the GV of human cadavers.

With the modelization used in this work, blood flow in any vein is determined by the pressure drop between its extremities, being directed toward the end with the lowest pressure. If the pressure difference between the vein ends reverts, the flow becomes retrograde. This general mechanism explains the cases of reflux found through our simulations, as explained for the intracranial, vertebral and azygos areas hereafter.

**Fig. 7:**
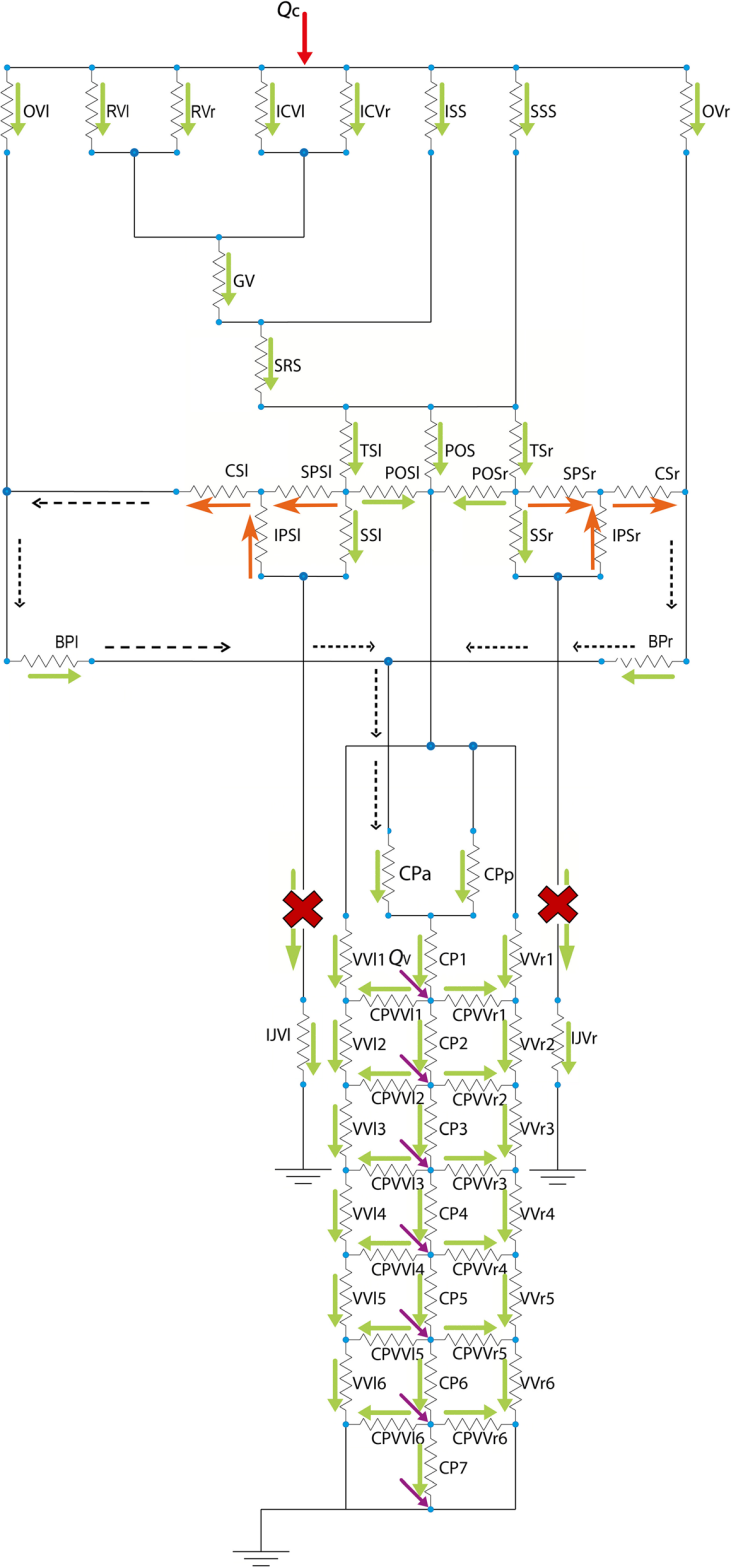
Flow directions computed by the model for the cerebral area with severe IJVs diameter reductions. The CS, IPS and SPS flows are inverted (orange arrows) compared to their physiologic direction shown in Fig. 2. In physiologic conditions, these intracranial veins drain into the IJVs (Fig. 2). With severe IJVs stenoses, their flow is reduced or blocked; an alternative drainage pathway for the CS, IPS and SPS blood flow is highlighted with dotted arrows

The upstream of one IJV is more influenced by its diameter change than intracranial vessels: its pressure begins to increase for small IJV diameters reduction. When both the IJVs are stenotic, our model predicted retrograde flow for the intracranial vein proximal to the IJVs themselves, in particular for the CS, IPS and SPS. Figure 5 represents how the flow inversion occurs in the IPS_l_. The pressures of its two ends were plotted over progressive reductions in IJV diameter (Fig. 5a): since they are superimposed on each other, it is clearly visible when one exceeds the other, i.e., for severe bilateral IJVs stenosis. Together with this pressure drop inversion, the IPS_l_ flow inverted its direction (Fig. 5b). The preferred drainage path of CS, IPS and SPS in this case was the anterior cervical plexus (Fig. 7) instead of the IJVs (Fig. 2), since it offers lower resistance to flow. The presence of collateral vessels, as modeled in this work, did not prevent intracranial pressure increase and reflux. Indeed, one or more collateral vessels in parallel to a stenotic IJV have higher resistance to flow as compared to a patent IJV, even if the total cross sectional area is the same [19]. If a clearer picture was needed, the model could be tuned for a single patient-specific case by setting ad hoc geometry, number, position and connections of collaterals. By doing this, it could be tested how a wider or higher number of collateral vessels would guarantee enough drainage and lower intracranial pressures, avoiding intracranial vessels reflux. Future work involving this kind of simulation could provide a better understanding about how collateral flow impacts intracranial pressure. Indeed, collateral number, dimensions and their anastomosis to the IJV are variable and are fundamental as an alternative pathway in clinical cases of IJV obstruction [27, 28].

The VV morphological obstructions had a low impact on the intracranial region, but a high impact on the pressure of the cervical and vertebral areas, besides on the areas connecting them to the intracranial vessels. When the pressure increase was higher in the upper portion of the VVs than in the intracranial region, a retrograde flow could occur in the posterior occipital sinuses. The blood of the last vessels was redirected to the IJVs instead of flowing to the CPp and VVs.

For a patent azygos, the pressure of its distal tracts is higher than that of the proximal ones: this is visible at Fig. 6a, for diameter reduction = 0 %. The flow is directed from its distal to its proximal tracts, to the superior vena cava, as shown in Fig. 2. The simulation of the proximal azygos obstruction produced asevere pressure increase (Fig. 6a, pressure between AZY4 and AZY5 and between AZY5 and AZY6). Conversely, the pressures of the distal districts were almost unaffected by the change (Fig. 6a, pressure between AZY11 and AZY12). When the obstruction caused an excess of pressure in the proximal compared to the distal part of an azygos portion, the pressure gradient changed its direction. The blood flow reverted its physiologic direction according to the pressure gradient. This can be observed in Fig. 6b, which shows how the azygos flow rate changes when proximal azygos obstruction is simulated. The AZY5 flow becomes retrograde when its distal pressure (between AZY5 and AZY6) exceeds the proximal one (between AZY4 and AZY5). For this particular case, this happens when the proximal azygos diameter is halved. The same can be observed for AZY11 flow, with less diameter reduction. A similar mechanism affected the thoracic plexus. The thoracic and azygos refluxes were drained to the right atrium through the inferior vena cava instead of the superior vena cava.

### Jugular vein obstructions: comparison of model simulations and real cases

Comparing our ECD data with the model estimates in the most prevalent clinical situation (the IJV obstruction), we found agreements in intracranial reflux topography. Comparing our ECD data with the model estimates in the most prevalent clinical situation (the IJV obstruction), we found agreements in intracranial reflux topography.

A quantitative comparison could not be done since we did not measure the IJV diameter during ECD acquisitions. However, the results of the model simulations have to be read as a proof of concept: the percentages of IJVs diameter reductions corresponding to intracranial reflux, or to intracranial pressure increase of at least 200 %, are not intended to be considered absolute thresholds. Indeed, they depend on the chosen “normal” IJVs diameter and other vessel dimensions. Regarding venous dimensions, a high inter-subject variability is described in literature, with different physiological vessels dimensions and flow rates among the studies, and a high deviation from the mean within each study [30, 32, 44, 49, 58].

Furthermore, the venous reflux detected in clinical cases often involves only a portion of the vessel and can be intermittent [42]. This fact can be explained by observing the occurrence of separated flow regions in a single vessel, as studied with a Lagrangian approach to fluid flow analysis [59, 60]. Conversely, with the present kind of modelization, a vessel can be classified as refluent if its flow rate is completely reverted. Local flow separation cannot be described through our approach. However, considering the relatively low values of Reynolds' number (maximum = 715 in the SSS), we may reasonably infer that such peculiar flow structures wouldn't significantly affect the vessel resistance calculations in our model.

### Limits and perspectives

The results of our model and all the simulations provided by it have to be evaluated while taking into consideration the main limitations of our type of modelization: the model is steady-state and it is valid only for analyzing the supine position.

Regarding the subject position, a future work for studying a standing subject should consider the effect of atmospheric pressure on IJVs, which collapse, and the role of collateral pathways [28].

As to the steady-state limitation, this means that every pressure and flow of the network is not time-variant, so the modulation of the venous velocity due to the cardiac or the thoracic pump are neglected. A recent work [61] showed that the latter highly influences the venous return of the IJVs, especially for in the deep inspiration, because of the aspiration effect of the pleural cavity. However, the mean flow of the intracranial veins is less modulated by the respiratory phases. The minor effect of respiration and cardiac pulsations on intracranial vein flow was also shown through MRI with the analysis of the frequency components in the range of respiration and heart beat. The flattened flow rate waveform of cerebral sinus veins can be due to their fixed diameter and protective effect of the dura mater [31]. Moreover, the IJV cross sectional area changes over the cardiac cycle [62]. However, due to the high inter-subject variability in terms of IJV cross sectional areas and ranges of variation, the choice of considering an average constant value can be considered a reasonable approximation.

Another aspect to point out is that the refluxes observed in clinical cases could be due to the sum of the effects of many morphological alterations (i.e., other abnormalities besides IJV or VV or AZY obstruction), while we separately simulated the effects of the main routes obstruction, obtaining intracranial refluxes only for severe IJV stenoses. Nonetheless, it is possible to use this model to test the effect of many other morphological alterations. Owing to the linearity of the model, the effects of any combination of anatomical changes may be obtained through the superposition principle, i.e., by linearly combining the effects of single anatomical alterations. It is hence straightforward to adapt the model to patient-specific features.

## Conclusion

In the context of a wide scientific interest for the link between venous flow and CNS disorders, the use of this model for the simulation of specific patterns (i.e., pervious *vs* stenotic vessels) or venous variants provides the estimate of their impact on pressures and flow in the main intracranial vessels.

## Additional files

Additional file 1:
**Lengths and diameters of venous vessels included in the model.** The plexus was considered an equivalent measure, due to the fact these districts are composed by a network-like structure. *: these vessels were subsequently divided in segments, as explained in the text. **: for IJVs also a physiological diameter of 1 cm [29] and 0.73 cm [32] were considered, to evaluate intracranial pressure and reflux onset related to smaller IJVs reported in literature. Additional file 2:
**Results obtained with model simulations of internal jugular veins (IJVs) diameter reduction and physiologic IJV diameter reported in Gisolf et al. [** 29 **].** A. Pressure at the confluence of Rosenthal veins (RVs) to Galen vein (GV) *vs* internal jugular veins (IJVs) diameter reduction. Pressure doubled its initial value when both the IJV diameters reduced at least of 70 %. The result is symmetric for correspondent diameter reduction rates on the opposite side (not shown). B. Bars showing IJV_l_ upstream pressure increase versus IJVs diameter reduction. Pressure value doubled its initial value when IJV_l_ underwent a diameter reduction of at least 20 %. Additional file 3:
**Results obtained with model simulations of internal jugular veins (IJVs) diameter reduction and physiologic IJV diameter reported in Ciuti et al. [** 32 **].** A. Pressure at the confluence of Rosenthal veins (RVs) to Galen vein (GV) *vs* internal jugular veins (IJVs) diameter reduction. Pressure doubled its initial value when both the IJV diameters reduced at least of 60 %. The result is symmetric for correspondent diameter reduction rates on the opposite side (not shown). B. Bars showing IJV_l_ upstream pressure increase versus IJVs diameter reduction. Pressure value doubled its initial value when IJV_l_ underwent a diameter reduction of at least 20 %.

## References

[1] Zivadinov R, Chung CP (2013). Potential involvement of the extracranial venous system in central nervous system disorders and aging. BMC Med.

[2] D'haeseleer M, Cambron M, Vanopdenbosch L, De Keyser J (2011). Vascular aspects of multiple sclerosis. Lancet Neurol.

[3] Chung CP, Wang PN, Wu YH, Tsao YC, Sheng WY, Lin KN (2011). More severe white matter changes in the elderly with jugular venous reflux. Ann Neurol.

[4] Chung CP, Hu HH (2010). Pathogenesis of leukoaraiosis: role of jugular venous reflux. Med Hypotheses.

[5] Chung CP, Hsu HY, Chao AC, Sheng WY, Soong BW, Hu HH (2007). Transient global amnesia: cerebral venous outflow impairment-insight from the abnormal flow patterns of the internal jugular vein. Ultrasound Med Biol.

[6] Lochner P, Nedelmann M, Kaps M, Stolz E (2014). Jugular valve incompetence in transient global amnesia. A problem revisited. J Neuroimaging.

[7] Cejas C, Cisneros LF, Lagos R, Zuk C, Ameriso SF (2010). Internal jugular vein valve incompetence is highly prevalent in transient global amnesia. Stroke.

[8] Cheng CY, Chang FC, Chao AC, Chung CP, Hu HH (2013). Internal jugular venous abnormalities in transient monocular blindness. BMC Neurol.

[9] Chung CP, Hsu HY, Chao AC, Chang FC, Sheng WY, Hu HH (2006). Detection of intracranial venous reflux in patients of transient global amnesia. Neurology.

[10] Hsu HY, Chao AC, Chen YY, Yang FY, Chung CP, Sheng WY (2008). Reflux of jugular and retrobulbar venous flow in transient monocular blindness. Ann Neurol.

[11] Doepp F, Valdueza JM, Schreiber SJ (2008). Incompetence of internal jugular valve in patients with primary exertional headache: a risk factor?. Cephalalgia.

[12] Zamboni P, Galeotti R, Menegatti E, Malagoni AM, Tacconi G, Dall'Ara S (2009). Chronic cerebrospinal venous insufficiency in patients with multiple sclerosis. J Neurol Neurosurg Psychiatry.

[13] Beggs C, Chung CP, Bergsland N, Wang PN, Shepherd S, Cheng CY (2013). Jugular venous reflux and brain parenchyma volumes in elderly patients with mild cognitive impairment and Alzheimer's disease. BMC Neurol.

[14] Chung CP, Beggs C, Wang PN, Bergsland N, Shepherd S, Cheng CY (2014). Jugular venous reflux and white matter abnormalities in Alzheimer's disease: a pilot study. J Alzheimers Dis.

[15] Zivadinov R, Bastianello S, Dake M, Ferral H, Haacke EM, Haskal ZJ (2014). Recommendations for multimodal noninvasive and invasive screening for detection of extracranial venous abnormalities indicative of chronic cerebrospinal venous insufficiency: a position statement of the International Society for Neurovascular Disease. J Vasc Interv Radiol.

[16] Comi G, Battaglia MA, Bertolotto A, Del Sette M, Ghezzi A, Malferrari G (2013). Observational case–control study of the prevalence of chronic cerebrospinal venous insufficiency in multiple sclerosis: results from the CoSMo study. Mult Scler.

[17] Dolic K, Marr K, Valnarov V, Dwyer MG, Carl E, Hagemeier J (2011). Sensitivity and specificity for screening of chronic cerebrospinal venous insufficiency using a multimodal non-invasive imaging approach in patients with multiple sclerosis. Funct Neurol.

[18] Dolic K, Siddiqui AH, Karmon Y, Marr K, Zivadinov R (2013). The role of noninvasive and invasive diagnostic imaging techniques for detection of extra-cranial venous system anomalies and developmental variants. BMC Med.

[19] Zaniewski M, Simka M (2012). Biophysics of venous return from the brain from the perspective of the pathophysiology of chronic cerebrospinal venous insufficiency. Rev Recent Clin Trials.

[20] Beggs CB (2013). Venous hemodynamics in neurological disorders: an analytical review with hydrodynamic analysis. BMC Med.

[21] Caiazzo A, Montecinos G, Müller L, Haacke EM, Toro E (2015). Computational haemodynamics in stenotic internal jugular veins. J Math Biol.

[22] Lakin WD, Stevens SA, Tranmer BI, Penar PL (2003). A whole-body mathematical model for intracranial pressure dynamics. J Math Biol.

[23] Mynard JP, Smolich JJ (2015). One-dimensional haemodynamic modeling and wave dynamics in the entire adult circulation. Ann Biomed Eng.

[24] Ursino M, Lodi CA (1997). A simple mathematical model of the interaction between intracranial pressure and cerebral hemodynamics. J Appl Physiol (1985).

[25] Ambarki K, Baledent O, Kongolo G, Bouzerar R, Fall S, Meyer ME (2007). A new lumped-parameter model of cerebrospinal hydrodynamics during the cardiac cycle in healthy volunteers. IEEE Trans Biomed Eng.

[26] Bergsneider M, Alwan AA, Falkson L, Rubinstein EH (1998). The relationship of pulsatile cerebrospinal fluid flow to cerebral blood flow and intracranial pressure: a new theoretical model. Acta Neurochir Suppl.

[27] Zamboni P, Sisini F, Menegatti E, Taibi A, Malagoni AM, Morovic S (2013). An ultrasound model to calculate the brain blood outflow through collateral vessels: a pilot study. BMC Neurol.

[28] Gadda G, Taibi A, Sisini F, Gambaccini M, Zamboni P, Ursino M (2015). A new hemodynamic model for the study of cerebral venous outflow. Am J Physiol Heart Circ Physiol.

[29] Gisolf J, van Lieshout JJ, van Heusden K, Pott F, Stok WJ, Karemaker JM (2004). Human cerebral venous outflow pathway depends on posture and central venous pressure. J Physiol.

[30] Valdueza JM, Schmierer K, Mehraein S, Einhaupl KM (1996). Assessment of normal flow velocity in basal cerebral veins. A transcranial doppler ultrasound study. Stroke.

[31] Schaller B (2004). Physiology of cerebral venous blood flow: from experimental data in animals to normal function in humans. Brain Res Brain Res Rev.

[32] Ciuti G, Righi D, Forzoni L, Fabbri A, Pignone AM (2013). Differences between internal jugular vein and vertebral vein flow examined in real time with the use of multigate ultrasound color Doppler. AJNR Am J Neuroradiol.

[33] Cho YI, Kensey KR (1991). Effects of the non-Newtonian viscosity of blood on flows in a diseased arterial vessel. Part 1: Steady flows. Biorheology.

[34] Newton DG, Potts NT. Radiology of the Skull and Brain. 1st ed. St Louis: C.V. Mosby; 1974.

[35] Lambertini G. Anatomia umana. 1st ed. Padova (Italy): Piccin; 1978.

[36] Rhoton AL (2002). The cerebral veins. Neurosurgery.

[37] Felten DL, Shetty A. Atlante di neuroscienze di Netter. Milano (Italy): Elsevier srl; 2011.

[38] Zamboni P, Consorti G, Galeotti R, Gianesini S, Menegatti E, Tacconi G (2009). Venous collateral circulation of the extracranial cerebrospinal outflow routes. Curr Neurovasc Res.

[39] Doepp F, Hoffmann O, Schreiber S, Lammert I, Einhaupl KM, Valdueza JM (2001). Venous collateral blood flow assessed by Doppler ultrasound after unilateral radical neck dissection. Ann Otol Rhinol Laryngol.

[40] Bastianello S, Romani A, Viselner G, Tibaldi EC, Giugni E, Altieri M (2011). Chronic cerebrospinal venous insufficiency in multiple sclerosis: clinical correlates from a multicentre study. BMC Neurol.

[41] Laganà MM, Preti MG, Forzoni L, D'Onofrio S, De Beni S, Barberio A (2013). Transcranial ultrasound and magnetic resonance image fusion with virtual navigator. IEEE Trans Multimedia.

[42] Forzoni L, D'Onofrio S, Farina M, Semplici P, Corsi M, Furia R, et al. Combined Ultrasound Technologies and Optimized Probe Design for Neck Veins Examination. Proceedings of the IASTED Biomedical Engineering BioMed Congress; 2012. p. 252–9.

[43] Gideon P, Thomsen C, Gjerris F, Sorensen PS, Stahlberg F, Henriksen O (1996). Measurement of blood flow in the superior sagittal sinus in healthy volunteers, and in patients with normal pressure hydrocephalus and idiopathic intracranial hypertension with phase-contrast cine MR imaging. Acta Radiol.

[44] Mehta NR, Jones L, Kraut MA, Melhem ER (2000). Physiologic variations in dural venous sinus flow on phase-contrast MR imaging. AJR Am J Roentgenol.

[45] Park HK, Bae HG, Choi SK, Chang JC, Cho SJ, Byun BJ (2008). Morphological study of sinus flow in the confluence of sinuses. Clin Anat.

[46] Saiki K, Tsurumoto T, Okamoto K, Wakebe T (2013). Relation between bilateral differences in internal jugular vein caliber and flow patterns of dural venous sinuses. Anat Sci Int.

[47] Manara R, Mardari R, Ermani M, Severino M, Santelli L, Carollo C (2010). Transverse dural sinuses: incidence of anatomical variants and flow artefacts with 2D time-of-flight MR venography at 1 Tesla. Radiol Med.

[48] Alper F, Kantarci M, Dane S, Gumustekin K, Onbas O, Durur I (2004). Importance of anatomical asymmetries of transverse sinuses: an MR venographic study. Cerebrovasc Dis.

[49] Stoquart-Elsankari S, Lehmann P, Villette A, Czosnyka M, Meyer ME, Deramond H (2009). A phase-contrast MRI study of physiologic cerebral venous flow. J Cereb Blood Flow Metab.

[50] Talbert DG (2008). Raised venous pressure as a factor in multiple sclerosis. Med Hypotheses.

[51] Schlesinger B (1939). The venous drainage of the brain, with special reference to the galenic system. Brain.

[52] Schelling F (1986). Damaging venous reflux into the skull or spine: relevance to multiple sclerosis. Med Hypotheses.

[53] Wu X, Wu W, Wu J, Zhang HL (2014). Diagnosis: thrombosis of the vein of Galen. Ann Saudi Med.

[54] Zivadinov R, Magnano C, Galeotti R, Schirda C, Menegatti E, Weinstock-Guttman B (2013). Changes of cine cerebrospinal fluid dynamics in patients with multiple sclerosis treated with percutaneous transluminal angioplasty: a case–control study. J Vasc Interv Radiol.

[55] Magnano C, Schirda C, Weinstock-Guttman B, Wack DS, Lindzen E, Hojnacki D (2012). Cine cerebrospinal fluid imaging in multiple sclerosis. J Magn Reson Imaging.

[56] Beggs CB, Magnano C, Shepherd SJ, Belov P, Ramasamy D, Hagemeier J (2015). Dirty-appearing white matter in multiple sclerosis: volumetric MR imaging and magnetization transfer ratio histogram analysis. J Neuroimaging.

[57] Iencean SM, Poeata I, Iencean AS, Tascu A (2015). Cerebral venous etiology of intracranial hypertension and differentiation from idiopathic intracranial hypertension. PubMed - NCBI.

[58] Beards SC, Yule S, Kassner A, Jackson A (1998). Anatomical variation of cerebral venous drainage: the theoretical effect on jugular bulb blood samples. Anaesthesia.

[59] Simka M (2014). Chronic cerebrospinal venous insufficiency: current perspectives. J Vasc Diagn.

[60] Shadden SC, Taylor CA (2008). Characterization of coherent structures in the cardiovascular system. Ann Biomed Eng.

[61] Zamboni P, Menegatti E, Pomidori L, Morovic S, Taibi A, Malagoni AM (2012). Does thoracic pump influence the cerebral venous return?. J Appl Physiol.

[62] Sisini F, Tessari M, Gadda G, Di Domenico G, Taibi A, Menegatti E (2015). An ultrasonographic technique to assess the jugular venous pulse: a proof of concept. Ultrasound Med Biol.

