# Supplementary material for: An anatomy-based lumped parameter model of cerebrospinal venous circulation: can an extracranial anatomical change impact intracranial hemodynamics?
Source: BMC Neurol. 2015 Jun 23;15:95. doi: 10.1186/s12883-015-0352-y (PMC4476203; doi:10.1186/s12883-015-0352-y)
Supplement: Additional file 1: — Lengths and diameters of venous vessels included in the model. The plexus was considered an equivalent measure, due to the fact these districts are composed by a network-like structure. *: these vessels were subsequently divided in segments, as explained in the text. **: for IJVs also a physiological diameter of 1 cm [29] and 0.73 cm [32] were considered, to evaluate intracranial pressure and reflux onset related to smaller IJVs reported in literature. [file 12883_2015_352_MOESM1_ESM.docx]

**Additional file 1. Lengths and diameters of venous vessels included in the model.** The plexus was considered an equivalent measure, due to the fact these districts are composed by a network-like structure. *: these vessels were subsequently divided in segments, as explained in the text. **: for IJVs also a physiological diameter of 1 cm^33^ and 0.73 cm^34^ were considered, to evaluate intracranial pressure and reflux onset related to smaller IJVs reported in literature.

| ***Vessel*** | ***Length***  ***[cm]*** | ***Diameter***  ***[cm]*** |
| --- | --- | --- |
| Ophthalmic vein right (OV_r_) | 5.25 | 0.2 |
| Ophthalmic vein left (OV_l_) | 5.25 | 0.2 |
| Basal vein of Rosenthal right (R_r_) | 5 | 0.3 |
| Basal vein of Rosenthal left (R_l_) | 5 | 0.3 |
| Internal cerebral vein right (ICV_r_) | 3 | 0.2 |
| Internal cerebral vein left (ICV_l_) | 3 | 0.2 |
| Inferior sagittal sinus (ISS) | 9 | 0.1 |
| Superior sagittal sinus (SSS) | 19.22 | 0.45 |
| Great vein of Galen (GV) | 1.4 | 0.4 |
| Straight sinus (SS) | 3.96 | 0.17 |
| Transverse sinus right (TS_r_) | 5.25 | 0.88 |
| Transverse sinus left (TS_l_) | 5.25 | 0.88 |
| Posterior occipital sinus root (POS) | 5 | 0.15 |
| Posterior occipital sinus right (POS_r_) | 5 | 0.15 |
| Posterior occipital sinus left (POS_l_) | 5 | 0.15 |
| Anterior occipital sinus right (AOS_r_) | 5 | 0.15 |
| Anterior occipital sinus left (AOS_l_) | 5 | 0.15 |
| Cavernous sinus right (CS_r_) | 5.5 | 0.2 |
| Cavernous sinus left (CS_l_) | 5.5 | 0.2 |
| Superior petrosal sinus right (SPS_r_) | 3.96 | 0.11 |
| Superior petrosal sinus left (SPS_l_) | 3.96 | 0.11 |
| Inferior petrosal sinus right (IPS_r_) | 4.52 | 0.11 |
| Inferior petrosal sinus left (IPS_l_) | 4.52 | 0.11 |
| Sigmoid sinus right (SS_r_) | 12.25 | 0.53 |
| Sigmoid sinus left (SS_l_) | 12.25 | 0.53 |
| Internal jugular vein right (IJV_r_) | 15 | 1.7** |
| Internal jugular vein left (IJV_l_) | 15 | 1.7** |
| Collaterals | 15 | 0.4 |
| Vertebral vein right (VV_r_) | 15* | 0.6 |
| Vertebral vein left (VV_l_) | 15* | 0.6 |
| Cervical plexus (CP) | 17.5* | 0.18 |
| Connective vessels between cervical plexus and vertebral veins (CPVV) | 1.8 | 0.18 |
| Thoracic plexus (TP) | 30* | 0.18 |
| Azygos vein (AZ) | 30* | 1.0 |
| Connective vessels between thoracic plexus and azygos vein (TPAZ) | 1 | 0.18 |
| Inferior vena cava (CV, CV1-2 ) | 12.18* | 2.0 (1.0) |
| Lumbar plexus (LP) | 17.5* | 0.18 |
| Lumbar vein (LV) | 10.3* | 1.0 |
| Connective vessels between lumbar plexus and lumbar veins (LPLV) | 1 | 0.18 |
