# Supplementary figures and images for: An anatomy-based lumped parameter model of cerebrospinal venous circulation: can an extracranial anatomical change impact intracranial hemodynamics?
Source: BMC Neurol. 2015 Jun 23;15:95. doi: 10.1186/s12883-015-0352-y (PMC4476203; doi:10.1186/s12883-015-0352-y)

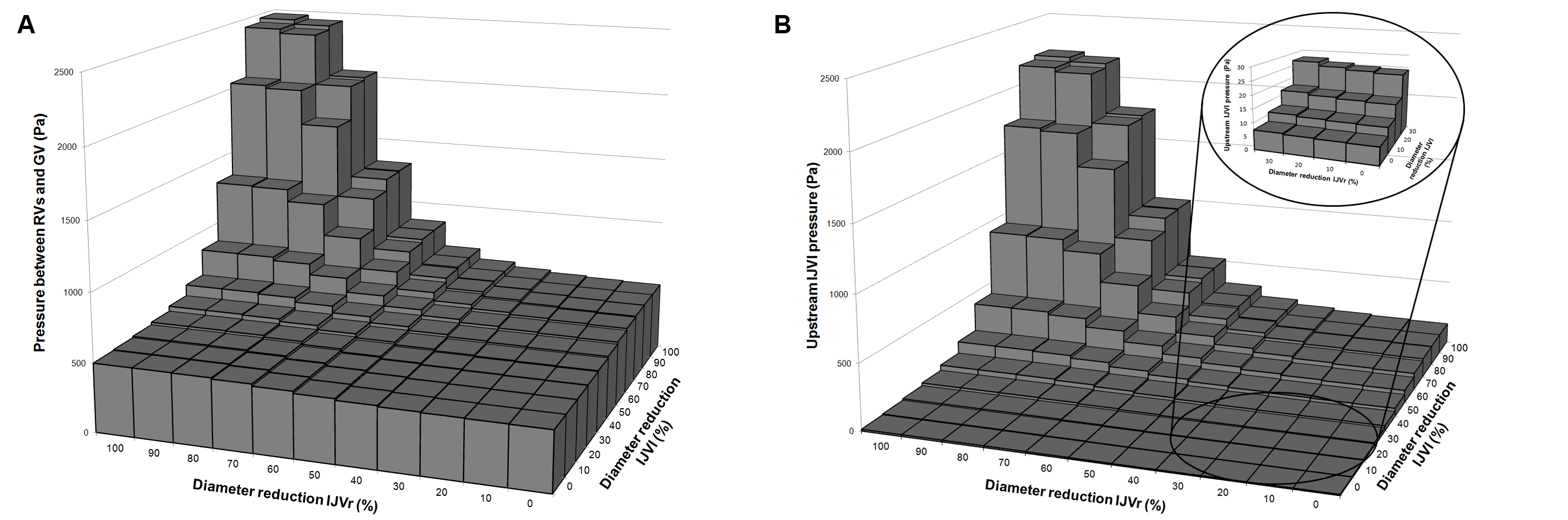

Supplement: Additional file 2: — Results obtained with model simulations of internal jugular veins (IJVs) diameter reduction and physiologic IJV diameter reported in Gisolf et al. [ 29 ]. A. Pressure at the confluence of Rosenthal veins (RVs) to Galen vein (GV) vs internal jugular veins (IJVs) diameter reduction. Pressure doubled its initial value when both the IJV diameters reduced at least of 70 %. The result is symmetric for correspondent diameter reduction rates on the opposite side (not shown). B. Bars showing IJVl upstream pressure increase versus IJVs diameter reduction. Pressure value doubled its initial value when IJVl underwent a diameter reduction of at least 20 %. [file 12883_2015_352_MOESM2_ESM.jpeg]

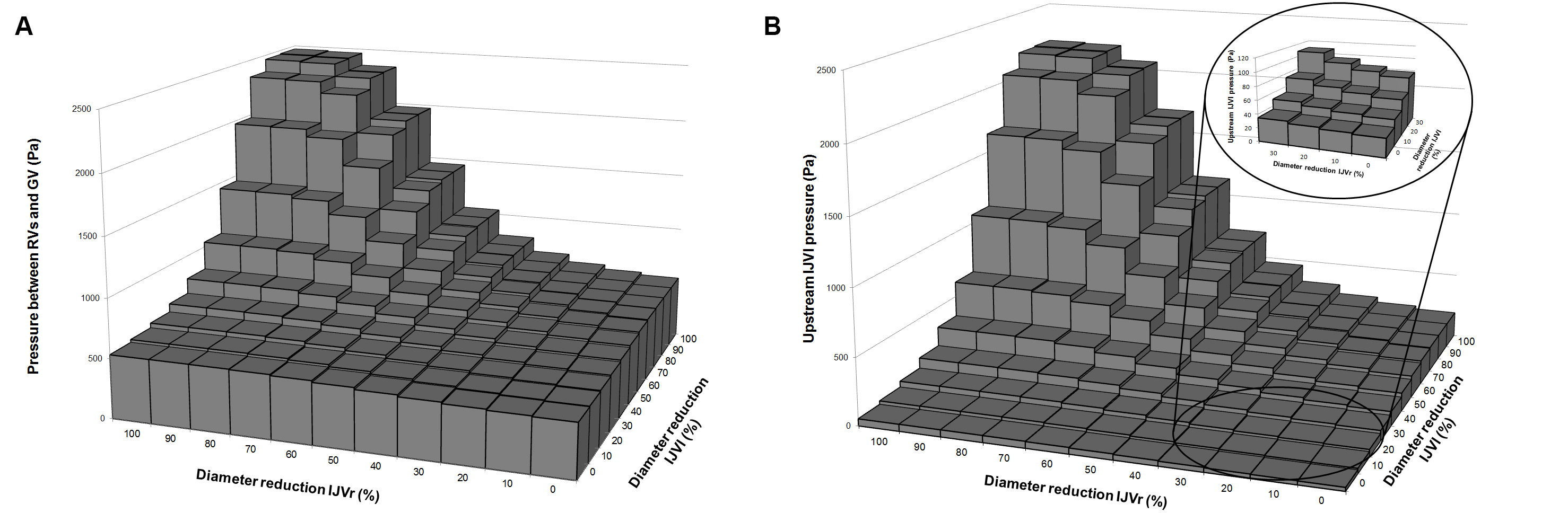

Supplement: Additional file 3: — Results obtained with model simulations of internal jugular veins (IJVs) diameter reduction and physiologic IJV diameter reported in Ciuti et al. [ 32 ]. A. Pressure at the confluence of Rosenthal veins (RVs) to Galen vein (GV) vs internal jugular veins (IJVs) diameter reduction. Pressure doubled its initial value when both the IJV diameters reduced at least of 60 %. The result is symmetric for correspondent diameter reduction rates on the opposite side (not shown). B. Bars showing IJVl upstream pressure increase versus IJVs diameter reduction. Pressure value doubled its initial value when IJVl underwent a diameter reduction of at least 20 %. [file 12883_2015_352_MOESM3_ESM.jpeg]
